# Blocking IL-19 Signaling Ameliorates Allergen-Induced Airway Inflammation

**DOI:** 10.3389/fimmu.2019.00968

**Published:** 2019-04-30

**Authors:** Yun-Han Weng, Wei-Yu Chen, Yen-Lin Lin, Jiu-Yao Wang, Ming-Shi Chang

**Affiliations:** ^1^Department of Biochemistry and Molecular Biology, College of Medicine, National Cheng Kung University, Tainan, Taiwan; ^2^Kaohsiung Chang Gung Memorial Hospital, Institute for Translational Research in Biomedicine, Kaohsiung, Taiwan; ^3^Institute of Microbiology & Immunology, College of Medicine, National Cheng Kung University, Tainan, Taiwan; ^4^Department of Pediatrics, College of Medical, National Cheng Kung University, Tainan, Taiwan

**Keywords:** interleukin-19, IL-20R1, asthma, airway inflammation, allergy

## Abstract

Asthma is a chronic inflammatory disease of the airway. Its major symptoms are reversible breathing problems causing airway narrowing and obstruction. IL-19 is a member of the IL-10 family cytokines. We previously showed that IL-19 induces T-helper 2 (Th2) cytokines and that asthma patients had higher serum IL-19 levels. To further examine whether inhibiting IL-19 and its receptor (IL-20R1) protected rodents against asthma, we used *Dermatophagoides pteronyssinus* (*Der p*; house dust mites) to induce chronic airway inflammation in wild-type C57BL/6 and IL-20R1-deficient mice and then analyzed the effect of the IL-20R1 deficiency on the pathogenesis of asthma. We also examined whether inhibiting IL-19 and IL-20R1 ameliorated *Der p*-induced chronic asthma. *Der p* induced IL-19 in lung airway epithelial cells, type 2 alveolar cells, and alveolar macrophages. An IL-20R1 deficiency abolished IL-19-induced Th2 cell differentiation *in vitro*. Th2 cytokine expression, immune cell infiltration in the bronchoalveolar lavage, airway hyperresponsiveness (AHR), and bronchial wall thickening were lower in *Der p*-challenged IL-20R1-deficient mice. Anti-IL-20R1 monoclonal antibody (mAb) 51D and IL-19 polyclonal antibody (pAb) both ameliorated *Der p*-induced AHR, lung immune cell infiltration, bronchial wall thickening, and Th2 cytokine expression. Moreover, we confirmed that anti-IL-19 mAb (1BB1) attenuated lung inflammation in a rat ovalbumin-induced asthma model. This is the first report to show that inhibition of IL-19 by targeting IL-19 or IL-20R1 protected rodents from allergic lung inflammation. Our study suggests that targeting IL-19 signaling might be a novel therapeutic strategy for treating allergic asthma.

## Introduction

Asthma is a chronic inflammatory disease of the airways characterized primarily by T-helper 2 (Th2) lymphocyte-mediated immune responses and associated with bronchial hyperresponsiveness, airflow obstruction, and airway remodeling (1). The global incidence of asthma is increasing, and it leads to significant use of healthcare resources (2). The protease activity of two major group-1 allergens (*Dermatophagoides pteronyssinus* [*Der p*] 1 and *Dermatophagoides Farinae* [*Der f* ] 1) from house dust mites (HDMs), two of the most important allergens worldwide, causes barrier dysfunction, induces the production of proinflammatory cytokines in epithelial cells, and induces Th2 responses (3–5).

The importance of type 2 immune responses, characterized by the production of interleukin-4 (IL-4), IL-5, and IL-13, has received much attention in the pathogenesis of asthma (6, 7). IgE was the first successful biological target used in patients with allergic disease and asthma (8). Recently, therapies targeting IL-4, IL-5, and IL-13 have shown potential efficacy for treating asthma (9). Although the majority of asthma patients benefit from current commercial therapies to control the symptoms, some patients do not respond well to these therapies (9). Thus, new asthma therapies that can inhibit not only airway hyper-responsiveness (AHR), but also mucus hyper-secretion and variable airflow obstruction, are needed.

IL-19 is a member of the IL-10 family, which includes IL-10, IL-19, IL-20, IL-22, melanoma differentiation-associated gene (MDA)-7 (IL-24), and AK155 (IL-26) (10, 11). IL-19 binds to IL-20 receptor (R)1/IL-20R2, a heterodimer complex mediating its signal transduction, and an activator of transcription (STAT)3 (12). IL-19 is produced primarily by monocytes, in which lipopolysaccharide (LPS) and granulocyte macrophage colony-stimulating factor (GM-CSF) upregulate IL-19 expression (13). Treating monocytes with IL-19 stimulates IL-6 and tumor necrosis factor (TNF)-α expression and induces monocyte apoptosis and the production of reactive oxygen species (ROS) (14). IL-19 is involved in inflammatory diseases such as rheumatoid arthritis (15), kidney injury (16), psoriasis (17), and breast cancer (18), and induces angiogenesis in endothelial cells (19). Acutely induced IL-19 in systemic inflammation promotes neutrophil chemotaxis and causes lung injury in mice undergoing endotoxin shock (20).These together suggest the potential roles of IL-19 as a tissue-derived inflammatory mediator.

We previously reported higher IL-19 expression in asthma patients and that patients with high IL-19 expression also have high IL-4 and IL-13 expression (21). We also found that IL-19 upregulated IL-13 and IgE production in asthmatic mice and that IL-19 induced Th2 cytokines *in vitro*, which suggested that IL-19 is substantially involved in allergic asthma (21). However, little is known about the molecular mechanism of IL-19 signaling in the pathogenesis of asthma. In this study, we investigated the role of IL-19 in asthmatic animal models in order to determine the therapeutic potential of IL-19-signaling antagonists for treating allergic asthma.

## Materials and Methods

### Animals

All animal experiments were done using the protocols of the National Institutes of Health standards and guidelines for the care and use of experimental animals. The research procedures were approved by the Animal Ethics Committee of National Cheng Kung University (IACUC NO. 107272 and 105077).

### Generating IL-20R1 Knockout Mice

IL-20R1 knockout^(−/−)^ mice were generated and maintained on a C57BL/6 genetic background, as previously described (22). Female wild-type (WT) C57BL/6 and IL-20R1^−/−^ mice were used for the *Der p*-induced-asthma model and for the *in vitro* Th2 differentiation experiments.

### *Der p*-Induced Asthmatic Mouse Model

The allergen *Der p* (1 g of lyophilized whole body extract in diethyl ether [Allergon, Engelholm, Sweden]) was dissolved in pyrogen-free isotonic saline, filtered with a 0.22-μm filter, and stored at −80°C before it was used (23). The LPS concentration of the *Der p* preparations was <0.96 endotoxin U/mg/*Der p* (E-Toxate [*Limulus* amebocyte lysate] test kit; Sigma-Aldrich, St. Louis, MO, USA). Groups of specific pathogen-free, 6–8 week-old C57BL/6 female mice (Laboratory Animal Center, National Cheng Kung University, Tainan, Taiwan) were intranasally (i.n.) inoculated with *Der p* (10 μl: 2.5 mg/ml) for 10 days. Control mice were inoculated with saline instead of with *Der p*. Seventy-two hours after the last *Der p* inoculation, the mice were killed, and blood samples were collected. Lung tissue was removed from control mice and asthmatic mice, and bronchoalveolar lavage fluid (BALF) was isolated and analyzed for immune cell infiltration. For antibody neutralization experiments, control isotype mIgG (6 mg/kg), 51D (6 mg/kg), pre-immune rabbit IgG, or IL-19 pAb were given 1 h after *Der p* treatment on day 0, 2, 4, 6, 8, and 10.

### Measuring Airway Resistance and Hyperresponsiveness

Mice were anesthetized, steel cannulae were inserted into their tracheas, and then they were individually placed in a chamber to measure, using the Buxco FinePointe system [Data Sciences International (DSI), St. Paul, MN, USA], their lung resistance (R_L_) while they were exposed to increasing doses of acetyl-β-methylcholine chloride (methacholine; Sigma-Aldrich, St. Louis, MO, USA). Dynamic airway resistance (Penh value) was noninvasively measured using unrestrained whole body plethysmography (Buxco Electronics, Wilmington, NC, USA) while they were exposed to increasing aerosol concentrations of methacholine.

### Histology and Immunohistochemistry

Lung tissues were embedded in paraffin, cut into 4 μm sections, and stained with hematoxylin and eosin (H&E). Inflammatory cell infiltration and lung architecture were assessed by light microscopy. The mucus secretion level was detected by periodic acid-Schiff (PAS; Sigma-Aldrich) staining. Lung sections were deparaffinized, hydrated in water, and then stained with periodic acid for 5 min. For immunohistochemistry, samples were paraffin embedded, sectioned, and stained by standard immunohistochemistry methods as previously described (24, 25). An additional antigen retrieval step was applied by heating samples in a Tris-based buffer (pH 9.0) to 95°C for 20 min. After blocking in 5% bovine serum albumin for 30 min at room temperature, tissues were incubated overnight at 4°C with primary antibodies against IL-19 (1:100; ab14106, Abcam), IL-20R1 (1:100; ab203196, Abcam), IL-20R2 (1:100; ab95824, Abcam), and SPC (1:100; ab211326, Abcam). The immune-reactivity of positive staining was developed by using the AEC chromogen kit (Romulin AEC Chromogen Kit; Biocare Medical, Walnut Creek, CA) and counterstained with Mayer's hematoxylin (J. T. Baker, Phillipsburg, NJ). Images were captured using an immunofluorescence microscope (Olympus BX51, Tokyo, Japan) or scanned using 3D Histech Pannoramic MIDI (3DHISTECH).

### Lung Histopathology

The degree of lung inflammation was determined as described previously (26–28). Serial sections (4 μm) were stained with hematoxylin and eosin (H&E), Alcian blue/periodic acid-Schiff, and Picro Sirius Red (Abcam). Three to four randomly selected H&E-stained lung tissue sections per group were analyzed per sample for quantification of lung inflammation scores. The tissue inflammation score was evaluated by two independent pathologists who were blinded to the sample codes as previously described (28): 0, no inflammatory cell infiltration; 1, little inflammatory cell infiltration; 2, 1 layer of inflammatory cells around the airway; 3, 2–4 layers of inflammatory cells around the airway; 4, 4 or more layers of inflammatory cells around the airway. For quantification of bronchial wall thickness, the thicknesses of airway walls from 5 to 10 random airways per slide were measured by using Olympus Viewer 3 image software (Olympus, Tokyo, Japan). For quantification of airway fibrosis, the percentage of Sirius Red-stained area in 5–10 random airways per slide (high power field) was measured by using ImageJ v1.48 (NIH, USA).

### Generating Anti-mIL-19 Polyclonal Antibody

Mouse (m) IL-19 polyclonal antibody (pAb) was generated using the standard procedure to immunize rabbits with mIL-19 recombinant protein. After the rabbits had been immunized, serum samples were collected and anti-IL-19 pAb was purified using IL-19-bound affinity chromatography. Antibodies purified from rabbit pre-immune serum using protein A chromatography (GE Healthcare, Illinois, USA) were used as control antibodies.

### Antibody Preparation

Anti-IL-19 monoclonal antibody (mAb) (1BB1) was prepared as previously described (21). Anti-IL-20 receptor (IL-20R1) mAb (51D) was generated using standard protocols as previously described (29).

### Analyzing IL-19, Stem Cell Factor, and Thymic Stromal Lymphopoietin Expression in Epithelial Cells

A549 and BEAS-2B cells (ATCC, Manassas, VA, USA) were plated for 12 h in Dulbecco's Modified Eagle's Medium (DMEM) with 10% fetal bovine serum. Epithelial cells were kept in serum-free DMEM for 8 h, and then incubated with *Der p* (500 ng/ml, Allergon, Engelholm, Sweden) or IL-19 (200 ng/ml) for 24 h. The culture media was collected, and stem cell factor (SCF), IL-19, and thymic stromal lymphopoietin (TSLP) protein levels were analyzed using enzyme-linked immunosorbent assay (ELISA). Mouse SCF and mouse TSLP ELISA kits were purchased from eBioscience, San Diego, USA. Mouse IL-19 protein was detected by homemade sandwich ELISA as previous described (21).

### Detecting Cytokines in Serum, BALF, and Lung Tissue Lysates

Protein levels of IL-4 (50-1128931, eBioscience), IL-5 (88-7054-22, eBioscience), IL-9 (50-112-5217, eBioscience), IL-13 (88-7137, eBioscience), SCF, TSLP, IgE, and IgA (88-50450, Invitrogen) in serum, BALF, lung tissue lysates, and culture medium were detected using ELISA kits according to manufactures' instructions. For analysis of protein levels in lung tissues, 50 μg of tissue lysates were analyzed by using the following ELISA kits: IL-33 (DY3626, R&D Systems), IL-17 (DY421, R&D Systems), CCL11 (DY420, R&D Systems), IL-22 (DY582, R&D Systems), and Amphiregulin (DY989, R&D Systems).

### *In vitro* Activity of IL-19 on CD4^+^ T Cells

Single-cell suspensions were prepared from splenocytes depleted of red blood cells (RBCs). CD4^+^ T cells were subsequently isolated to >97% purity with positive selection using anti-CD4 (L3T4) magnetic beads (Miltenyi Biotec, Auburn, CA, USA) (21). For *in vitro* differentiation assays, CD4^+^ T cells were incubated for 2 days with 1 μg/ml of plate-bound anti-CD3 and 1 μg/ml of plate-bound anti-CD28 mAbs with IL-2 (20 U/ml), IL-4 (10 ng/ml), anti-IL-12 mAb (1 μg/ml), and anti-interferon (IFN)-γ Ab (10 μg/ml) for Th2 differentiation. Three days after the primary incubation, the cells were washed by PBS and then incubated with or without IL-19 (200 ng/ml) added in the Th2 differentiation medium containing IL-2 (20 U/ml), IL-4 (10 ng/ml), anti-IL-12 mAb (1 μg/ml), and anti-interferon (IFN)-γ Ab (10 μg/ml) for 7 days. Fresh cytokines and Abs were added every 2 days. On day 7, cell culture supernatants were collected for ELISA of Th2 cytokine production (IL-5, IL-9, IL-13, and IL-4). All the cytokines and Abs used were purchased from PeproTech, Rocky Hill, NJ, USA. ELISA kits for human cytokines were purchased from eBiosciences.

### Ovalbumin-Induced Asthmatic Rat Model

Ovalbumin (OVA) (Sigma-Aldrich) was prepared in pyrogen-free PBS and precipitated at a 1:1 ratio with Al(OH)_3_ (Sigma-Aldrich). Six 6- to 8-week-old female Sprague-Dawley rats (250–300 g) were sensitized with 2 mg of OVA (1 ml of OVA-Al(OH)_3_ suspension) given intraperitoneally on day 0, 7, and 14. Another 6 rats were given sham immunizations using PBS. Twenty-five days after the rats had been sensitized, for 5 days they were intranasally challenged daily with 1% OVA in 100 μl of PBS. Experimental rats (*n* = 6 per group) were given 1BB1 (10 mg/kg) and control rats were given isotype mouse immunogammaglobulin G1 (mIgG1) (10 mg/kg) 1 h before each intranasal OVA challenge. The rats were sacrificed on day 30 and the BALF and lung tissues were collected for histological analysis and mRNA preparation for qRT-PCR analysis.

### Quantitative Real-Time Polymerase Chain Reaction

Total RNA was isolated using Trizol reagent (Invitrogen, Carlsbad, CA, USA) followed by reverse transcription to generate cDNA (PrimeScript RT-PCR kit; Clontech, Palo Alto, CA, USA). The expression of transcripts was analyzed using polymerase chain reaction (PCR) with gene-specific primers. Glyceraldehyde phosphate dehydrogenase (GAPDH) was used as an internal control. The following sequence-specific primers were used in real-time PCR: hIL-19, F: 5′-CGC GGA TCC ACT CAG GAG ATG TCT GAT TTC-3′, R: 5′-AGC TGA GAA CAT TAC TTC ATG A-3′; hIL-20R1, F: 5′-AAC GCT TCT ATC CTT TCT TGG A-3′, R: 5′-GAT GAC TTT AGC CTT CCA TGC-3′; hIL-20R2, F: 5′-GTG CAC CTA GAA ACC ATG GA-3′, R': CCA TCT TCC AGA CGG AGA G-3′; hHPRT, F: 5′-TGA CCA GTC AAC AGG GGA CA-3′, R: 5′-TGC CTG ACC AAG GAA AGC AA-3′; rat IL13, F: 5′-CCA CAG GAC CCA GAG GAT ATT GA-3′, R: 5′-TAG CGG AAA AGT TGC TTG GAG TAA-3′; rat IL19, F: 5′-GCC ATG CAA ACT AAG GAC ACC-3′, R: 5′-TTG GTC ATG CAG CAC ACA TC-3′; rat GAPDH: F: 5′-ACA TGC CGC CTG GAG AAA CCT-3′, R: 5'-TCC ACC ACC CTG TTG CTG TAG-3′. The expression levels were amplified using a thermal cycler (LightCycler 480; Roche Diagnostics, Indianapolis, IN, USA) with SYBR Green I (Roche, Basel, Switzerland) for quantitative analysis normalized with GAPDH as an input control. Relative changes in mRNA expression were determined by calculating 2^−ΔΔ*Ct*^.

### Statistical Analysis

Prism 5.0 (GraphPad Software, San Diego, CA, USA) was used for all statistical analyses. The one-way analysis of variance (ANOVA) non-parametric test (Kruskal-Wallis test) or student *t*-test was used to compare the data between groups. *Post hoc* comparisons were done using Dunn's multiple comparison test. Results are mean ± standard deviation (SD). Significance was set at *P* < 0.05.

## Results

### IL-19 Expression Was Induced in *Der p*-Treated Lung Epithelial Cells

**Figure 1:**
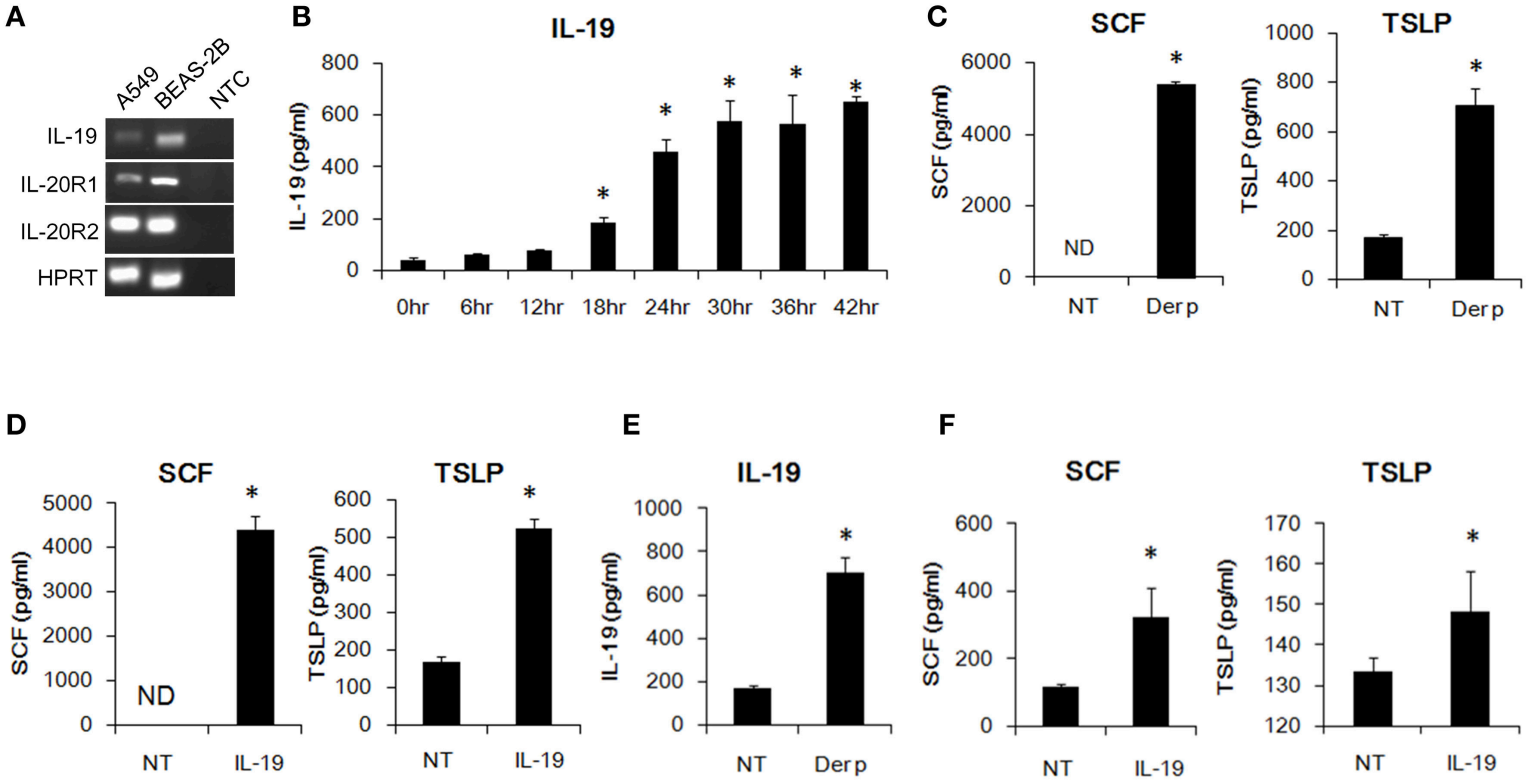
IL-19 was induced by *Der p* and upregulated SCF and TSLP production in lung epithelial cells *in vitro*. **(A)** RT-PCR analysis for the transcripts of IL-19, IL-20R1, and IL-20R2 in A549 and BEAS-2B cells. **(B)** IL-19 protein levels in the media of *Der p* (500 ng/ml)-treated A549 cells for different time courses. ^*^P < 0.05 by one-way non-parametric ANOVA. **(C)** Stem cell factor (SCF) and thymic stromal lymphopoietin (TSLP) protein levels in the culture media of A549 cells treated with *Der p* (500 ng/ml) for 24 h. **(D)** A549 cells were treated with IL-19 (200 ng/ml) for 24 h and the culture media were collected for ELISA analysis of SCF and TSLP levels. **(E)** IL-19 levels in the culture media from BEAS-2B cells treated with *Der p* (500 ng/ml) for 24 h. **(F)** SCF and TSLP protein levels in the culture medium from BEAS-2B cells treated with IL-19 (200 ng/ml) for 24 h. NT, no template control; ND, not detectable. ^*^P < 0.05 by *t*-test.

Bronchial epithelial cells are critical for the pathogenesis of asthma. HDM allergens cause bronchial constriction in asthma patients and induce an inflammatory response in the lungs because of the expression of cytokines, chemokines, and some inflammatory mediators (30, 31). We first confirmed whether bronchial epithelial cells express the transcripts of IL-19 and its receptor subunits, IL-20R1 and IL-20R2. Using RT-PCR, we found that human A549 lung epithelial cells and human BEAS-2B bronchial epithelial cells expressed IL-19 and its receptors subunits (Figure 1A). We next tested whether *Der p* induces IL-19 production *in vitro*. We co-cultured human A549 lung epithelial cells with *Der p* and collected the medium for ELISA analyses. IL-19 expression was induced in *Der p*-stimulated A549 cells (Figure 1B) in a time-dependent manner.

### SCF and TSLP Protein Expression Was Higher in *Der p*-Treated Lung Epithelial Cells *in vitro*

In addition to IL-19, we also detected higher SCF and TSLP protein levels in *Der p*-treated A549 cells than in control cells (Figure 1C). Epithelial-derived SCF supports the development and survival of mucosal mast cells at the airway surface (32). Allergens are processed by myeloid dendritic cells, which are conditioned by the TSLP secreted by epithelial cells and mast cells to attract Th2 cells (33, 34). To test whether IL-19 alone activates lung epithelial cells and induces cytokine production, we treated the A549 cells with IL-19 and collected the media for ELISA analysis. SCF and TSLP protein was induced in IL-19-treated A549 cells (Figure 1D). Similarly, IL-19 was induced by *Der p* and upregulated SCF, and TSLP production in the BEAS-2B lung epithelial cells *in vitro*. (Figures 1E,F). These data together showed that *Der p* induced IL-19 production and that IL-19 activated lung epithelial cells to produce SCF and TSLP *in vitro*.

### IL-20R1-Deficiency Attenuated Th2 Cell Differentiation *in vitro*

**Figure 2:**
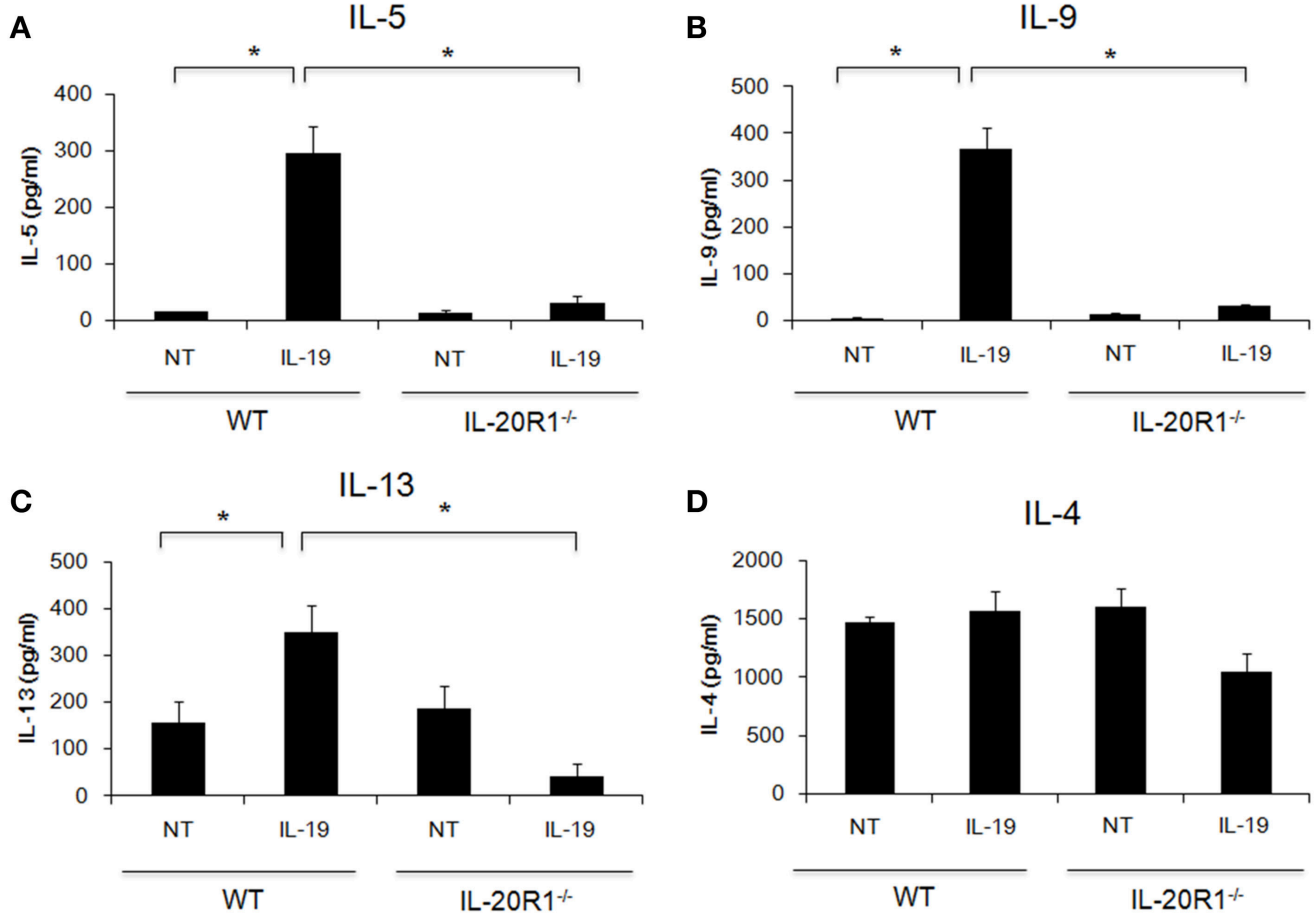
Deficiency of IL-20R1 attenuated IL-19-induced Th2 cell polarization *in vitro*. A negative selection of CD4^+^ T cells were isolated from the spleens of wild-type C57BL/6 (WT) and IL-20R1^−/−^ mice and then incubated on a plate coated with anti-CD3 and anti-CD28 monoclonal antibodies (mAbs), and then the CD4^+^ T cells were incubated in Th2-differentiaion condition with PBS or IL-19 (200 ng/ml) for another 7 days as described in the methods section. Conditioned medium was collected on day 7, and **(A)** IL-5, **(B)** IL-9, **(C)** IL-13, and **(D)** IL-4 expression levels were analyzed using ELISA. The expression of T-helper 2 (Th2) cytokines on IL-19-treated CD4^+^ T cells was upregulated. Data are the average of duplicate samples. NT, not treated (PBS controls). ^*^P < 0.05 by one-way non-parametric ANOVA.

*Der p* induces proinflammatory cytokines in epithelial cells and induces Th2 responses (3–5). The importance of type 2 immune responses in the pathogenesis of asthma has received much attention (6, 7). We previously reported that IL-19 promoted Th2 polarization *in vitro* (21). We next hypothesized that IL-19 signaling through the IL-20R1/IL-20R2 receptor complex promotes Th2 differentiation from naïve T cells and induces Th2 cytokine production. To test this hypothesis, we isolated CD4+ naïve T cells from WT and IL-20R1^−/−^ mice and differentiated the cells into Th2 phenotypes with and without IL-19 treatment (Figure 2). We collected and analyzed the culture media from the differentiated Th2 cells on day 7. IL-19 upregulated the production of IL-5 (Figure 2A), IL-9 (Figure 2B), and IL-13 (Figure 2C), but not IL-4 (Figure 2D), in differentiated Th2 cells from WT mice. IL-20R1 deficiency abolished IL-19-induced IL-5, IL-9, and IL-13 production in IL-19-treated CD4+ naïve T cells, which suggested that IL-20R1-mediated IL-19 signaling is crucial for IL-19-induced Th2 polarization *in vitro*.

### IL-19 Expression Was Upregulated in *Der p*-Induced Allergic Asthmatic Mice

**Figure 3:**
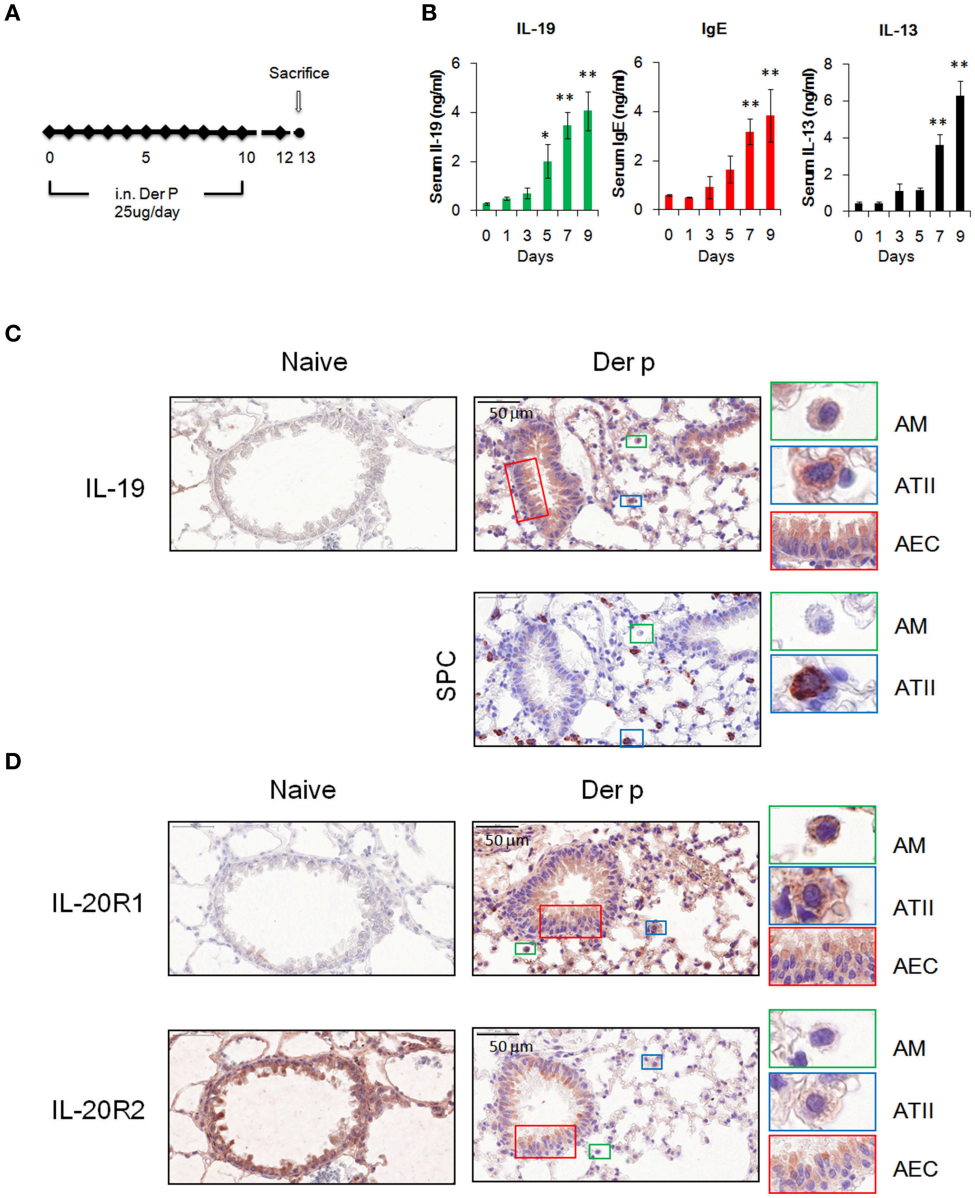
Expression of IL-19 and IL-19 receptors in lung tissues. **(A)** C57BL/6 mice were intranasally (i.n.) challenged with *Der p* extract (25 μg/mouse/day) for 10 days to induce asthma. **(B)** Dynamic serum levels of IL-19, IL-13, and immunoglobulin E (IgE) in *Der p*-sensitized mice (*n* = 5). ^*^P < 0.05, ^**^P < 0.01 compared with day 0 control by one-way non-parametric ANOVA. **(C)** Lung tissues were collected on day 13 and paraffin-sectioned for IHC staining for IL-19. Detection of the expression of surfactant protein C (SPC) in adjacent slide was used as marker for type II alveolar cells. **(D)** IHC staining for the expression of IL-20R1 and IL-20R2 with specific antibodies. Nuclei were counterstained with hematoxylin. Bars represented 50 μm. AM, alveolar macrophages; ATII, type II alveolar cells; AEC, airway epithelial cells.

We next examined whether IL-19 is involved in the pathogenesis of HDM-induced allergic asthma. Chronic asthmatic mice were induced by repeated intranasal administration of *Der p* for 10 days (Figure 3A). The serum IL-19 and IgE levels were higher after 5 days of *Der p* treatment (Figure 3B). Serum IL-13, a well-known critical mediator in the pathogenesis of asthma (35), was upregulated after day 7 (Figure 3B). This result indicated that *Der p*-induced allergic lung inflammation associated with the elevation of IL-19, IL-13, and IgE serum levels, and that IL-19 was induced earlier (at day 5) than the upregulation of IL-13 after *Der p* challenges.

### Upregulation of IL-19 and IL-19 Receptors in Lung Tissues From *Der p*-Treated Mice

To further confirm the source and target of IL-19 *in vivo*, we performed immunohistochemical staining to detect the protein expression of IL-19, IL-20R1, and IL-20R2 with specific antibodies. IL-19 expression was not detected in naïve lung tissues (Figure 3C). In lung tissues from *Der p* challenged mice, IL-19 was detected in alveolar macrophages (AM) that were distributed the alveolar space and in surfactant protein C (SPC)-producing type II alveolar cells (ATII). IL-19 was also upregulated in the airway epithelial cells (AEC) (Figure 3D). The expression of IL-20R1 receptor subunit was upregulated in AM, ATII, and AEC following *Der p* challenge, and minimal to undetectable levels were found in normal lung tissues (Figure 3D). In contrast to the induction of IL-19 and IL-20R1 by *Der p*, basal level of IL-20R2 expression was detected in AM, ATII, and AEC in the lungs from healthy and *Der p*-treated mice (Figure 3D). Taken together, the results of IHC staining revealed that the lung epithelial cells (AEC and ATII) and AM could be the sources and target cells of IL-19 in the context of *Der p*-induced lung inflammation.

### IL-20R1 Deficiency Protected the Mice Against *Der p*-Induced Chronic Asthma

**Figure 4:**
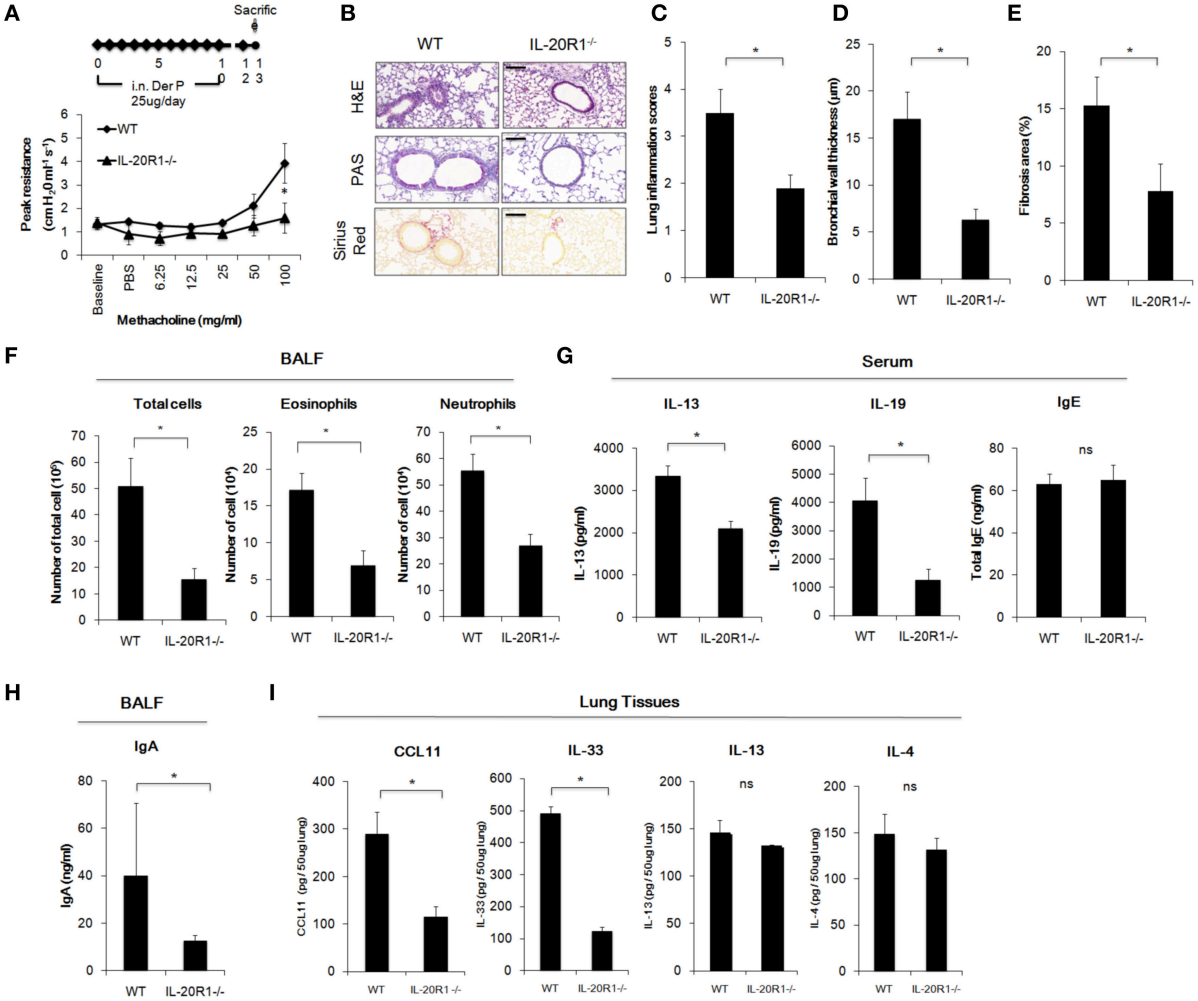
*Der p*-induced chronic asthma was attenuated in IL-20R1^−/−^ mice. Wild-type C57BL/6 and IL-20R1^−/−^ mice (*n* = 6 in each group) were intranasally (i.n.) challenged with *Der p* extract (25 μg/mouse/day) for 10 days to induce asthma. **(A)** Airway hyperresponsiveness (AHR) was analyzed on day 13. IL-20R1^−/−^ mice had lower peak airway resistance with increasing doses of methacholine than did wild-type mice. **(B)** Histopathology assessments for lung inflammation, airway wall thickness by Hematoxylin and Eosin (H&E) and PAS staining. Degree of collagen deposition was evaluated by Sirius Red staining. Bars represented 50 μm. **(C)** Quantification of lung inflammation scores. **(D)** Quantification of bronchial wall thickness. **(E)** Quantification of Fibrosis area. **(F)** The number of infiltrated immune cells in mouse bronchoalveolar lavage fluid (BALF). **(G)** Serum IL-13, IL-19, and IgE levels. **(H)** BALF IgA levels. **(I)** Inflammatory cytokine levels (CCL11, IL-33, IL-13, and IL-4) in the lung tissues. ^*^P < 0.05 by *t*-test. ns, not significant.

IL-20R1 and IL-20R2 could form functional receptor complex for IL-19 (12). Since IL-19 and IL-20R1 were induced in the lung following *Der p* challenge, it was hypothesized that IL-19 signaling via IL-20R1 axis contributes to the progression of lung inflammation. To verify the role of IL-20R1-mediated IL-19 signaling in the development of allergic lung inflammation, we compared the pathological outcomes between WT and IL-20R1^−/−^ mice in the *Der p*-induced chronic asthma model. The airway hyperresponsiveness (AHR) was lower in IL-20R1^−/−^ mice than in WT mice, indicating improved lung function in IL-20R1^−/−^ mice (Figure 4A). Histopathological analysis by using H&E, PAS, and Sirius Red staining were performed to evaluate the lung structure changes, pulmonary inflammation, and collagen deposition in the lung tissues (Figure 4B). The degree of lung inflammation (Figure 4C), bronchial wall thickening (Figure 4D), and airway fibrosis (Figure 4E) were lower in IL-20R1^−/−^ mice than in WT mice (Figures 4B–E). In addition, IL-20R1^−/−^ mice treated with *Der p* had fewer immune cells in their BALF than did WT mice (Figure 4F). The serum levels of IL-13 and IL-19 were lower in IL-20R1^−/−^ mice than in WT mice (Figure 4G). The serum IgE level, however, was not altered by IL-20R1-deficiency in *Der p*-challenged mice (63.09 ± 4.85 ng/ml in WT vs. 65.07 ± 7.01 ng/ml in IL-20R1^−/−^ mice, respectively) (Figure 4G). The IgA levels, which also associated with asthma onset (36), were lower in the BALF from IL-20R1^−/−^ mice than from WT mice (Figure 4H). We analyzed the levels of pro-inflammatory cytokines associated with immune cell infiltration and type 2 immune responses and found that CCL11 (37), and IL-33 (38) levels were lower in lung tissues of IL-20R1^−/−^ mice than in WT mice (Figure 4I). Levels of IL-13 and IL-4 were also slightly lower in IL-20R1^−/−^ but were not statistically significantly different (Figure 4I). We also analyzed the protein levels of IL-5, IL-17, IL-22, and Amphiregulin, and we did not find significant differences on the expression of these proteins between groups (data not shown). These data suggested that IL-20R1 deficiency protected the mice from *Der p*-induced chronic asthma and reduced proinflammatory cytokines associated with immune cell infiltration.

### Anti-IL-20R1 mAb (51D) Reduced the Severity of Asthma in Asthmatic Mice Model

**Figure 5:**
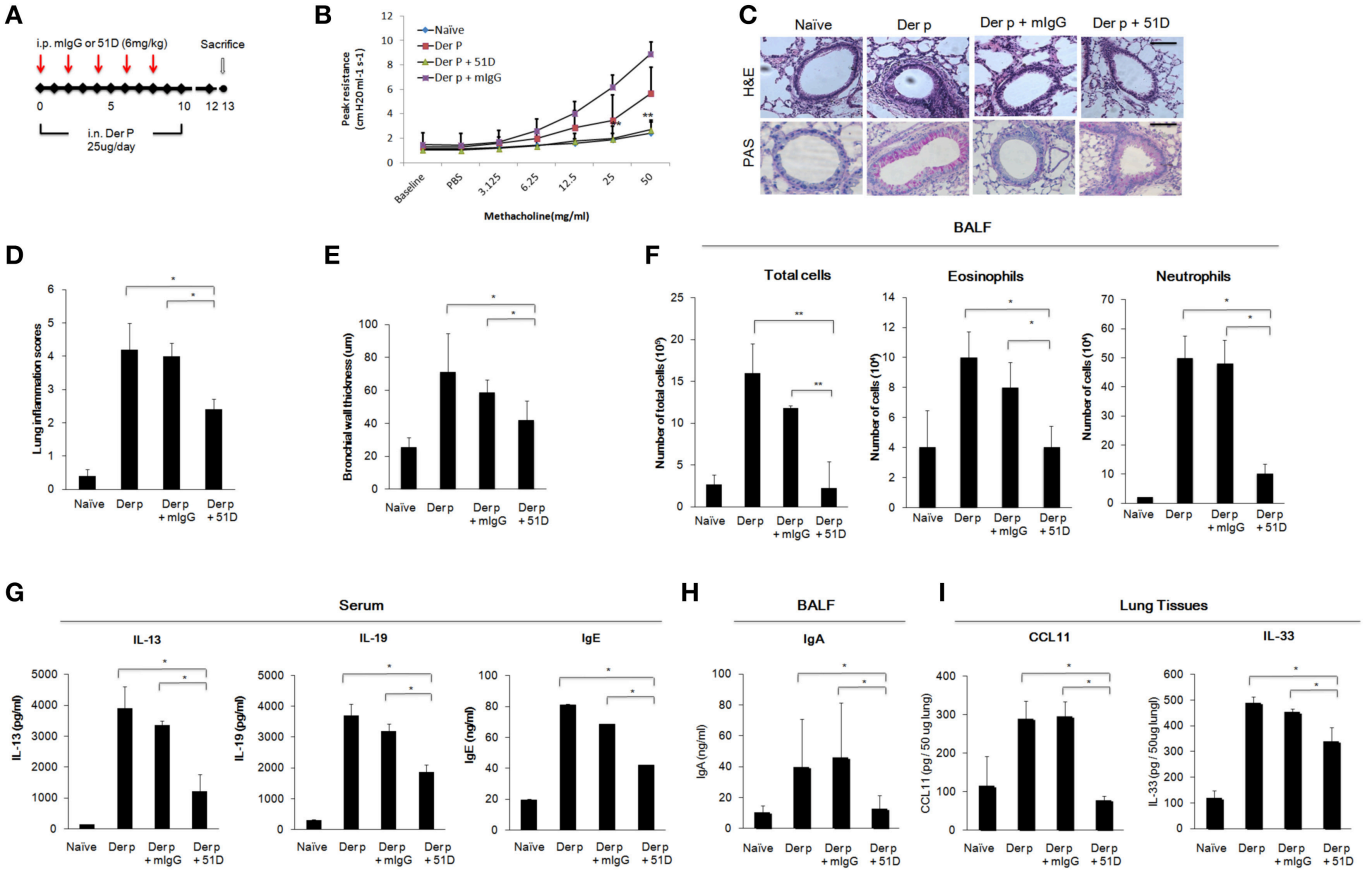
Anti-IL-20R1 mAb (51D) reduced airway inflammation in *Der p*-induced asthmatic mice. **(A)** Protocol of *Der p*-induced asthmatic mouse model with anti-IL-20R1 mAb (51D) treatment. C57BL/6 mice were intranasally (i.n.) challenged with *Der p* extract (25 μg/mouse/day) for 10 days to induce asthma. 51D (6 mg/kg) or control mIgG (6 mg/kg) was intraperitoneally injected into the *Der p*-sensitized mice on day 0, 2, 4, 6, and 8 (*n* = 6 per group). **(B)** AHR was analyzed in the mice 3 days after the last *Der p* treatment. Peak AHR was methacholine dose-dependently lower in 51D-treated mice. **(C)** Hematoxylin and eosin (H&E) staining showed less airway thickening and periodic acid-Schiff (PAS) staining showed fewer goblet cell areas in 51D-treated mice. **(D)** Lung inflammation scores of the mice. **(E)** Bronchial wall thickness. **(F)** Number of infiltrated immune cells, eosinophils, and neutrophils in mouse BALF. **(G)** Serum IL-13, IL-19, and IgE levels in the mice. **(H)** BALF IgA levels. **(I)** CCL11 and IL-33 levels in the lung tissues. ^*^P < 0.05, ^**^P < 0.01 by one-way non-parametric ANOVA.

We next examined whether 51D, an IL-20R1 blocking mAb previously shown to inhibit liver fibrosis in mice model (29), would also protect *Der p*-treated mice from developing chronic asthma by preventing lung inflammation. In this model, 51D or control mIgG were given intraperitoneally (i.p.) on day 0, 2, 4, 6, and 8 (Figure 5A). The AHR of the mice was measured on day 13. The AHR of the 51D-treated mice was significantly lower compared with *Der p*-treated or *Der p* plus mIgG groups (*p* < 0.05) (Figure 5B). H&E and PAS staining showed reduced lung inflammation, bronchial wall thickening, and mucus-secreting goblet cell areas in 51D-treated mice (Figures 5C–E). There were also fewer infiltrated immune cells, eosinophils, and neutrophils in the BALF of 51D-treated mice than in the BALF of Control group mice (Figure 5F). ELISA analysis showed significantly lower serum IL-13, IL-19, and IgE levels, as well as lower BALF IgA levels in 51D-treated mice (Figures 5G,H). Tissue protein levels of CCL11 and IL-33 were also lower in 51D-treated group (Figure 5I). These data together demonstrated that IL-20R1 neutralizing mAb 51D ameliorated *Der p*-induced allergic lung inflammation *in vivo*.

### *Der p*-Induced Chronic Asthma Was Attenuated in mIL-19 pAb-Treated Mice

**Figure 6:**
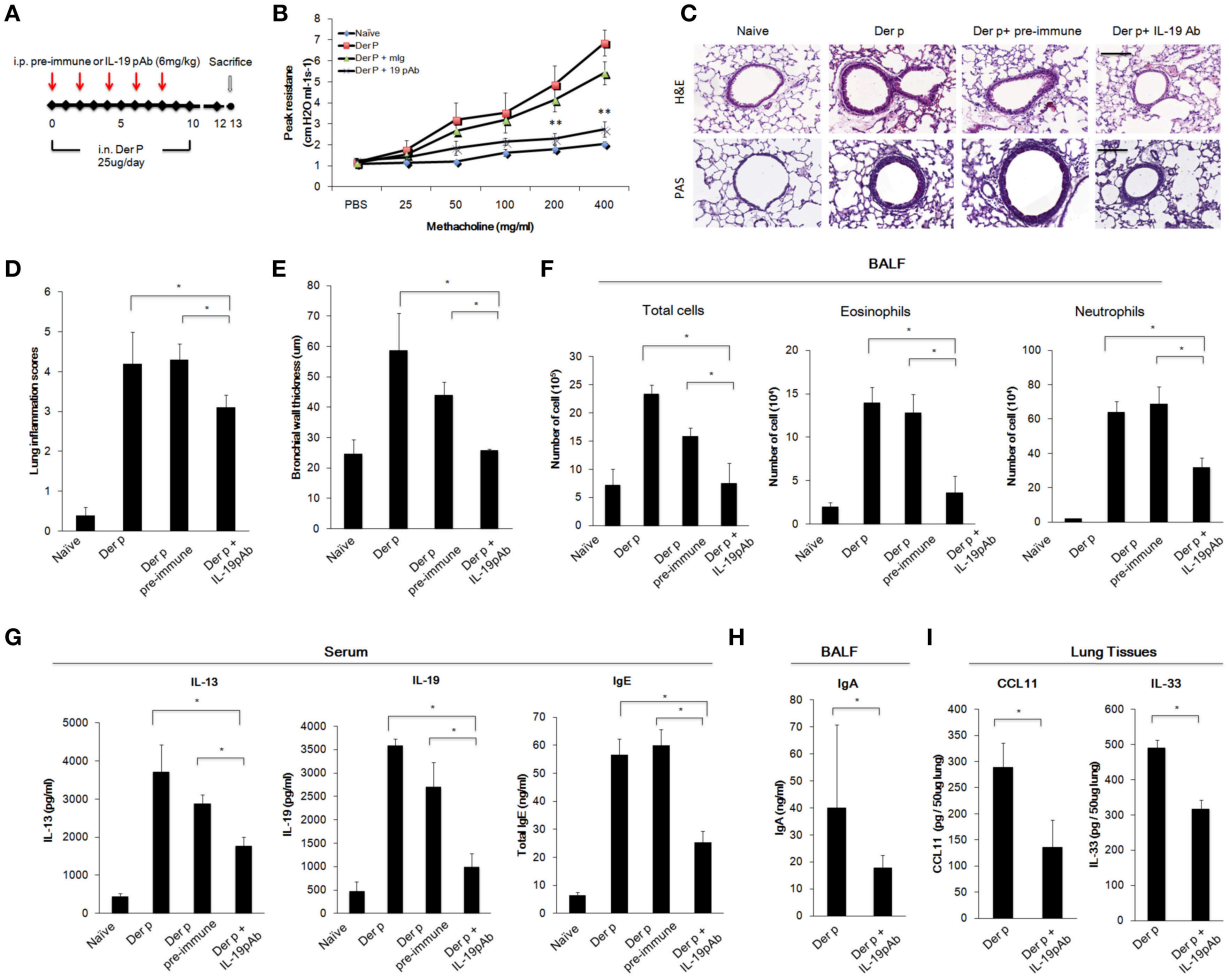
Anti-IL-19 antibody attenuated *Der p*-induced chronic asthma. **(A)** Protocol of *Der p*-induced asthmatic mouse model with anti-IL-19 pAb treatment. C57BL/6 mice were intranasally (i.n.) challenged with *Der p* (25 μg/mice/day) for 10 days to induce chronic airway inflammation. Anti-IL-19 pAb (6 mg/kg) or a control pre-immune IgG (6 mg/kg) was intraperitoneally injected into the *Der p* sensitized mice (*n* = 6 in each group). **(B)** AHR was analyzed in the mice 3 days after the last *Der p* treatment. Peak AHR was methacholine dose-dependently lower in 51D-treated mice. **(C)** Hematoxylin and eosin (H&E) staining showed reduced airway thickening and PAS staining showed fewer goblet cell areas in anti-IL-19 pAb-treated mice. **(D)** Lung inflammation scores of the mice. **(E)** Airway wall thickness. **(F)** Number of infiltrated immune cells, eosinophils, and neutrophils in mouse BALF. **(G)** Serum IL-13, IL-19, and IgE levels in the mice. **(H)** BALF IgA levels. **(I)** CCL11 and IL-33 levels in the lung tissues. ^*^P < 0.05, ^**^P < 0.01 by one-way non-parametric ANOVA.

To further confirm whether direct inhibition of IL-19 would attenuate chronic asthma *Der p*-treated mice, we treated mice with mIL-19 neutralizing antibodies. We previously generated the anti-human IL-19 mAb 1BB1, which showed specific neutralization activity against human IL-19 in a mouse model of breast cancer (39, 40), but 1BB1 did not effectively neutralize mouse IL-19 protein *in vivo* (data not shown). Thus, we generated rabbit polyclononal antibodies against mIL-19 and treated the *Der p*-induced asthmatic mice with the anti-mIL-19 pAb. The pre-immune serum-derived pAb was used as control (Figure 6A). AHR analysis showed that IL-19 blockade improved lung function in the *Der p*-challenged mice (Figure 6B). H&E staining revealed that IL-19 inhibition also reduced the degree of lung inflammation and the levels of bronchial wall thickening (Figures 6C–E). In addition, the number of infiltrated immune cells, eosinophils, and neutrophils in their BALF (Figure 6F), and the serum levels of IL-13, IL-19, IgE (Figure 6G), BALF IgA (Figure 6H), and lung tissue CCL11 and IL-33 levels (Figure 6I) were significantly lower in mIL-19 pAb-treated group than those of pre-immune pAb treated control group mice. These data together indicated that inhibition of IL-19 signaling through blocking IL-19 or IL-20R1 were both effective to attenuate *Der p*-induced pulmonary inflammation in the mouse model.

### Airway Inflammation Was Less Severe in Ovalbumin-Induced Asthmatic Rats Treated With Anti-IL-19 mAb 1BB1

**Figure 7:**
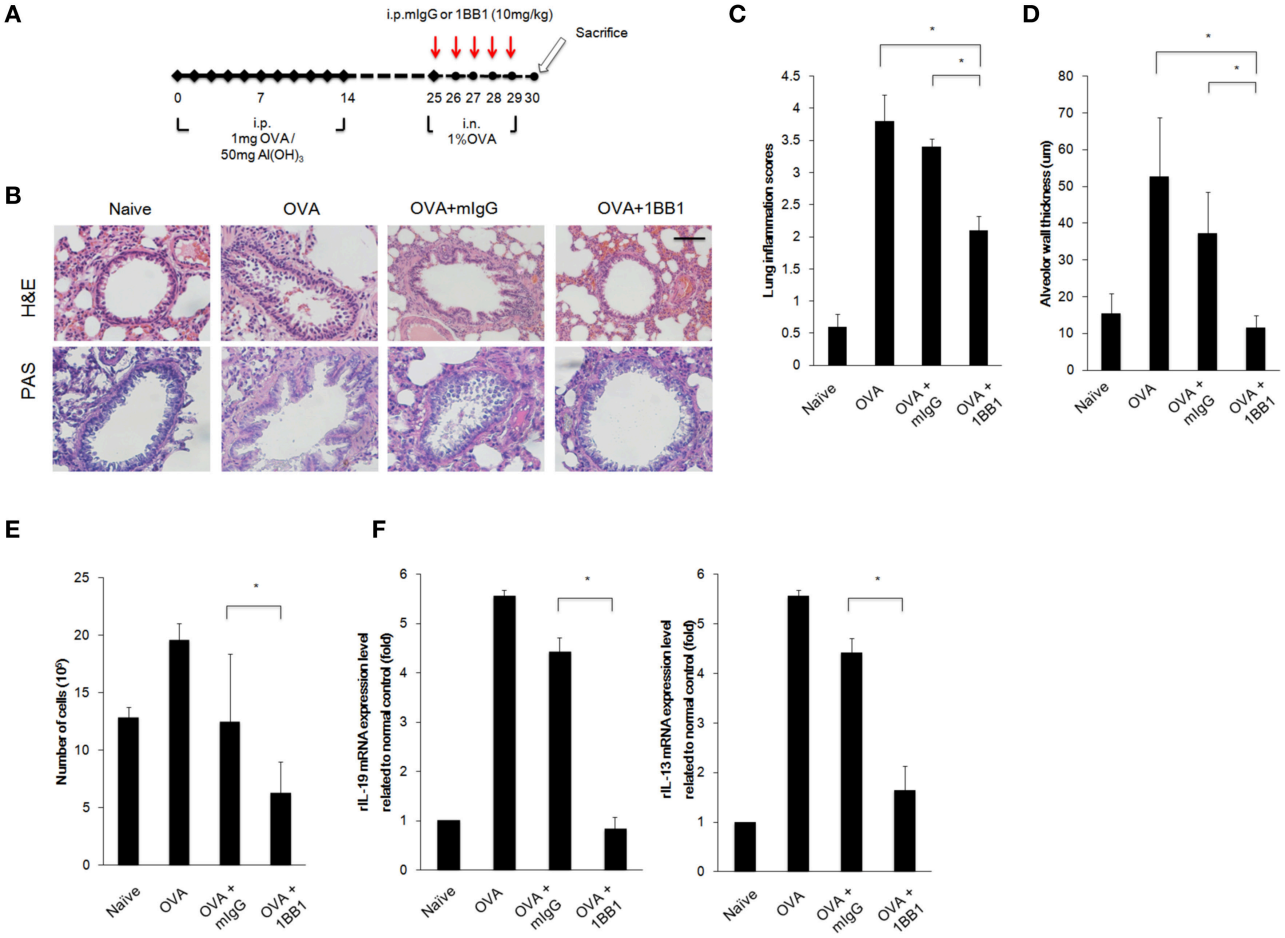
OVA-induced asthma was ameliorated in 1BB1-treated rats. **(A)** Protocol of ovalbumin (OVA)-induced asthmatic mouse model with anti-IL-19 mAb (1BB1) treatment. Wild-type Sprague-Dawley rats (*n* = 6 per group) were intraperitoneally (i.p.) injected with 1 mg of OVA adsorbed on 50 mg of gelatinous Al(OH)_3_ on days 0, 7, and 14. The rats were treated intranasally (i.n.) daily with 1% OVA in 100 μL of PBS from day 25 for 5 days. They were then dosed via i.p. injection with 1BB1 (10 mg/kg) or immunogammaglobulin (mIgG) (10 mg/kg) (control) 1 h before OVA treatment. **(B)** H&E and PAS staining showed less airway thickening in 1BB1-treated1 asthmatic rats. **(C)** Lung inflammation scores of the mice. **(D)** Airway wall thickness. **(E)** Number of infiltrated immune cells in rat BALF. **(F)** qRT-PCR analysis for the mRNA levels of IL-13 and IL-19 in rat lung tissue. ^*^P < 0.05 by one-way non-parametric ANOVA.

Our results demonstrated that targeting IL-19 or IL-20R1 could ameliorate *Der p*-induced asthma in the mouse model (Figures 5, 6). We thought to further confirm whether targeting IL-19 could ameliorate allergen-induced allergic airway inflammation in rats. In contrast to the use of *Der p* in mouse model, we established the asthmatic rat model by repeated immunization of rats with ovalbumin (OVA), which is derived from chicken eggs and is a frequently used allergen to induce a robust allergic pulmonary inflammation in laboratory rodents (41). In addition, our previous study demonstrated that our anti-human IL-19 mAb 1BB1 could cross-react with rat IL-19 and attenuated collagen-induced arthritis in rats (41). Thus, we tested whether IL-19 specific mAb 1BB1 would attenuate pulmonary inflammation in the OVA-induced asthma model in rats. To establish the rat asthmatic model, WT Sprague-Dawley rats were sensitized by repeated immunization of OVA 3 times on day 0, 7, and 14. The rats were then challenged by daily OVA intranasally from day 25 for 5 days to induce allergic lung inflammation (Figure 7A). For the antibody treatment groups, 1BB1 or control mIgG were intraperitoneally injected 1 h before OVA treatments (Figure 7A). The OVA-sensitized rats developed pathological airway inflammation outcomes similar to those in *Der p*-treated mice (Figure 7B). Histopathological analysis by H&E and PAS staining showed less lung inflammation and bronchial wall thickening and fewer mucus-secreting goblet cell areas in 1BB1-treated rats (Figures 7C,D). The number of infiltrated immune cells in their BALF (Figure 7E), and the mRNA levels of IL-19 and IL-13 (Figure 7F) in the lung tissues from 1BB1-treated rats were lower than in control group rats. These data together indicated that blocking IL-19 by polyclonal and monoclonal antibodies were effective to attenuate the pathological outcomes of allergen-induced asthma both in the mouse and rat models.

## Discussion

In this study, we established the mouse asthma models by repeatedly exposing the airways of mice to *Der p* and injecting rats with OVA and analyzed the mechanisms of the inflammatory responses. The *Der p*-induced asthma model was associated with increased AHR and bronchial wall thickening, elevated serum IL-19, IL-13, IgE levels, BALF IgA levels, increased immune cell infiltration, and tissue IL-33 and CCL11 in the lungs. Results from IL-20R1^−/−^ mice suggested that IL-19 signaling through IL-20R1 contributed to *Der p*-induced asthma. The IL-19 receptor has two subunits: IL-20R1 and IL-20R2. IL-20R2 is constitutively expressed in lung epithelium and other types of tissue (42). IL-20R1 is also expressed in lung epithelium; thus, lung epithelium could be the target of IL-19 (42). In this study, our results further confirmed that IL-20R2 was constitutively expressed while IL-19 and IL-20R1 were upregulated in the AM, ATII, and AECs in the lung tissues of mice with *Der p* challenge. These results together suggested that *Der p*-induced IL-19 could mediate lung inflammation via targeting to the cells expressing both of the IL-19 receptor subunits (IL-20R1 and IL-20R2) in the autocrine and paracrine manner.

IL-19 was shown to autoregulate IL-19 production *in vitro* (43). In the current study, *Der p*-induced IL-19 expression in A549 and BEAS-2B lung epithelial cells (Figure 1) and upregulated serum IL-19 levels in mice (Figure 3). In asthmatic mice, the degree of airway inflammation and IL-19 serum levels were lower in IL-20R1^−/−^ mice and 51D-treated mice, which suggested that IL-19 levels are associated with the outcomes of airway inflammation. The mechanism of the auto-amplification loop might explain why IL-19 levels were lower in the IL-20R1^−/−^ and 51D-treated mice. Autoregulation of IL-19, however, was not observed in IL-19-treated A549 cells (16), which likely imply that other immune cells, such as activated alveolar macrophages, contribute to the expression of IL-19 during airway inflammation.

In our mouse model, IL-20R1 deficiency reduced *Der p*-induced lung inflammation, bronchial wall thickening, and the levels of Th2 cytokines (IL-13, IL-33, CCL11), IgA, suggesting that IL-20R1 downstream signaling contribute to exacerbation of type 2 immune responses in the context of *Der p*-induced lung inflammation. We thus hypothesize that IL-20R1 is crucial for the effecter function of IL-19 to regulate T cell differentiation. *In vitro*, we confirmed that IL-19 promoted Th2 differentiation from CD4^+^ naïve T cells and that the Th2 differentiation was abolished in CD4^+^ T cells derived from IL-20R1 mice (Figure 2). *In vivo*, we found that using IL-20R1 mAb 51D to inhibit IL-20R1 attenuated *Der p*-induced asthma and Th2 cytokine expression. Additionally, IL-19 pAb treatment had a similar protective effect against *Der p*-induced pulmonary inflammation. These data together indicated that targeting IL-19 signaling might be a potential therapeutic strategy for treating or preventing the development of chronic asthma. IL-20, another IL-10 family cytokine, also binds to the IL-20R1/IL-20R2 receptor complex. However, the expression level of IL-20 was not altered after *Der p* treatment (data not shown), which suggested that IL-19 plays more dominant roles in IL-20R1-mediated allergic airway inflammation.

Allergic asthma is a hypersensitivity reaction initiated by immunologic mechanisms mediated by IgE antibodies (1). IgE is important for the initiation and propagation of the inflammatory cascade and allergic responses (9). Thus, IgE-mediated immunologic pathways have long been an attractive target for therapeutic agents in asthma and other allergic diseases (8). In the current study, we found that using antibodies to inhibit IL-19 or IL-20R1 in asthmatic mice lowered their serum IgE levels. This indicates that targeting IL-19 signaling might attenuate or even prevent the early development of airway remodeling and inflammation. Thus, IL-19 inhibitors might be novel and potent therapeutics for asthma because IL-19 likely mediates early allergic response after allergen exposure and attribute to the development of Th2-dominant airway inflammation. It is interesting to note that IL-20R1 deficiency did not reduce the serum IgE levels following *Der p challenge* compared with WT mice, which might attribute to higher basal serum IgE levels in naïve IL-20R1^−/−^ mice than did in WT mice (23.50 ± 16.4 ng/ml and 9.38 ± 3.64, respectively). Despite the unaltered serum IgE levels in IL-20R1^−/−^ mice upon *Der p* challenge, IL-20R1^−/−^ mice were more resistant to *Der p*-induced allergic airway inflammation compared to WT mice. An unidentified role of IL-20R1 downstream signaling on the regulation of IgE synthesis may exist in the context of genetic deficiency of IL-20R1 signaling and requires further investigation.

We previously reported that IL-19 upregulated IL-1β and TNF-α in A549 lung epithelial cells, which might promote an inflammatory response and cause pulmonary injury (16). Moreover, overexpression of IL-19 upregulated serum IL-13 levels only in asthmatic mice but not healthy mice, which suggested that IL-19-mediated allergic response might be dependent upon the tissue microenvironment (21). In the current study, we found that IL-19 induced lung epithelial cells to express TSLP, which is involved in allergic airway inflammation by activating group 2 innate lymphoid cells (ILC2) (38). TSLP released from injured epithelium after antigenic stimulation of allergic asthmatic airways is a key initiator of type-2 inflammation (38). It is possible that IL-19 activates ILC2 through TSLP and leads to elevated IL-13 levels. Our *in vivo* study also showed that the protein levels of IL-33 and CCL11 were reduced by IL-20R1 deficiency, IL-19 inhibition, or IL-20R1 blockades, suggesting that blocking IL-19 signaling inhibits allergic lung inflammation via reduction of the pro-inflammatory cytokines and reduced immune cell infiltration. IL-33 is also a key mediator for activation of ILC2 and eosinophils (38). CCL11 has been shown to promote eosinophil infiltration in allergen-induced lung inflammation (37). Although we did not analyze whether IL-19 could directly activate these immune cells (eosinophils, neutrophils, and ILCs), we did not exclude the possibility that IL-19 contributes to the amplification of type 2 immune responses via activation of the tissue-derived inflammatory mediators such as TSLP and IL-33. Whether IL-19 directly activates ILC2 and whether ILC2 activation is involved in IL-19-mediated airway inflammation require additional investigation. Comprehensive profiling of the direct target cells of IL-19 during the progression of allergic pulmonary inflammation is needed.

Rodent models of experimental allergic asthma have contributed greatly to our current understanding of the pathogenesis of this disease. In the present study, we established the *Der p-* or OVA-induced allergic airway inflammation models in mice and rats, respectively. The major difference between these two models is that allergens derived from HDMs exhibit intrinsic enzymatic protease activity and mimic the route of allergen exposure and allergic responses that are observed during human allergic airway inflammation (41). Conventional OVA-models, however, in rodents require co-administration of adjuvants to establish successful sensitization. The OVA model may not be the most disease-relevant model for allergic airway inflammation (44). The use of HDMs as allergens in rats is limited owing to the higher amount and cost of house dust mite preparation. Nevertheless, our data provided evidence that blocking IL-19 signaling may be a potential treatment strategy in these two commonly used preclinical rodent models of experimental allergic asthma.

In summary, this is the first report demonstrating that inhibition of IL-19 signaling by targeting IL-19 or IL-20R1 protected mice and rats from developing asthma in the rodent models. These findings provide new evidence that IL-19 is involved in the development of allergen-induced airway remodeling and inflammation and also indicates that IL-19 signaling is a promising target for anti-asthmatic intervention.

## Ethics Statement

This study was carried out in accordance with the recommendations of the National Institutes of Health standards and guidelines for the care and use of experimental animals. The protocol was approved by the Animal Ethics Committee of National Cheng Kung University (IACUC NO. 107272 and 105077).

## Author Contributions

J-YW and M-SC designed the study. Y-HW and Y-LL performed the experiments. Y-HW and W-YC analyzed the data. Y-HW, W-YC, and M-SC wrote the manuscript. W-YC and J-YW provided critical technical and scientific guidance and discussion.

### Conflict of Interest Statement

The authors declare that the research was conducted in the absence of any commercial or financial relationships that could be construed as a potential conflict of interest.
